# Reusable Sensor for Strontium Sulfate Scale Monitoring in Seawater

**DOI:** 10.3390/ma14030676

**Published:** 2021-02-01

**Authors:** Abdellatif Bouchalkha, Radouane Karli, Khalid Alhammadi

**Affiliations:** 1 Directed Energy Research Centre, Technology Innovation Institute, Abu Dhabi 9639, United Arab Emirates; 2 Electrical Engineering Department, Ecole Mohammedia des Ingenieurs (EMI), Mohammed V University-Agdal, Rabat 10090, Morocco; RadouaneKarli@research.emi.ac.ma; 3 Electrical and Computer Engineering Department, Khalifa University, Abu Dhabi 127788, United Arab Emirates; khalid.alhammadi@ku.ac.ae

**Keywords:** GHz spectroscopy, ions monitoring, strontium sulfate, scale monitoring, celestite, scaling sensor, reusability, microwave sensing, seawater, strontium ions

## Abstract

The onset of scaling in oil pipelines can halt or drastically reduce oil production, causing huge financial losses and delays. Current methods used to monitor scaling can take weeks, while the scaling process only takes few hours. The proposed sensor is designed for online monitoring of strontium ions concentration in seawater as an early scaling indicator. The sensor operates in the GHz range by probing the shift in the resonance frequency due to changes in the ionic concentrations of the medium. The results show selective sensitivity to changes in the strontium ions concentration even in the presence of many other ions found in seawater. The measured sensitivity is found to be stable and linear with a detection level of better than 0.08% (0.042 mol/L) of strontium ions in seawater. This work demonstrates a robust GHz sensor for strontium sulfate scale monitoring and early detection, which could be used in the oil industry to prevent huge production losses. These results could also be extended further to target the monitoring of other ions in different industrial sectors.

## 1. Introduction

During the past few years, microwave-sensing systems have grown rapidly in numbers and capabilities mainly due to their low-cost, easy fabrication and integration, small size, and non-invasive nature [1,2,3,4,5,6]. The sensing capabilities of the different microwave sensors mainly rely on the resonance principle and are attracting increasing interest in applications related to biosensing, microfluidic, environmental monitoring, and industrial applications in general. For example, they are used in thin-film detection and permittivity measurements [7,8], strain measurements in different directions [9], displacement measurements [10], detection of biomolecules [11], measurements of glucose concentration in water [12], and other sensing applications [13,14]. Most of these applications involve more complex designs with additional limitations related to the microfluidic applications. This integration between microwave technology and different fields is expected to lead to the development of new sensors with excellent performance. In fact, materials and methods conceived for the THz range are now also being investigated for several applications in the GHz range due to the strong potential for detection and sensing applications in medicine, health care, and environmental monitoring [4,15,16]. In particular, in the recent work in [16], they used filter paper as the substrate for their microstrip antenna to analyze body sweat. One of the limitations of this work was the single use of the sensor, and hence many sensors were required to complete this study. The sweat is absorbed by the filter paper, which cannot be used again, in addition to the cost involved in this process. To overcome these limitations, our proposed sensor uses different substrate materials with a more optimized design to ensure high sensitivity, repeatability, and reusability.

It is well known in the oil industry that strontium ions cause large amounts of scaling on the inner surface of the pipelines leading to a drastic reduction in oil production and huge financial losses [17,18,19,20]. One of the major challenges of the oil industry is to prevent scale development in the oil production pipelines [20,21,22,23,24,25]. This scaling is due to the mixing of injected seawater with the produced water from the well. The produced water contains many minerals depending on the type of rocks in the well and the geology of the region. The seawater, on the other hand, contains high concentrations of sulfate. The two waters are incompatible, and under certain levels of ions concentrations and specific thermodynamic conditions, a supper saturation of the mixed waters can be reached, leading to nucleation and immediate scale formation. This process is quite fast and unpredictable due to the many uncertainties and variations related to the produced underground water chemical composition and the varying high temperatures and high pressures in the oil wells [25].

According to the recent fieldwork [21] in Upper Zakum (4th largest oil field in the world) in Abu Dhabi, it was reported that the main scales present in the oil wells in this field are calcium carbonate (CaCO_3_) in the upper part of the wells and strontium sulfate (SrSO_4_) in the lower part of the wells. From the field data and records, the report clearly emphasized the big challenge faced in removing the strontium sulfate scale due to its hardness. In order to manage this challenge, they used a scale prediction software model to identify the wells with high scaling risk and monitor them closely. The model uses several parameters such as the scaling index, the ratio between seawater and produced water, and the chemical compositions of the water samples from the wells. This is an offline and very lengthy process that can take several weeks, while scaling can take just a few hours.

The fast process of scaling can, in fact, reduce oil production by more than 50% in only a few hours [23,24]. The consequences are drastic, with huge financial losses in production, repairs, maintenance, downtime, and even total damage to the well [21,22,23]. To prevent this, the current methods used are based on the injection of special chemicals or “scale inhibitors” [21,22,23]. These treatments are quite expensive and should only be used at a specific critical time based on certain guidelines related to the ionic composition of the water (scaling index).

Under specific temperature and pressure conditions, the mixing of seawater (rich in sulfate) with produced water (rich in strontium) can quickly lead to supersaturation and trigger strontium sulfate scale formation. Hence, the monitoring of the concentrations of strontium and sulfate ions are among the main indicators of strontium sulfate scaling.

In this paper, we propose and demonstrate the working of a GHz sensor based on a microstrip antenna designed to target the monitoring of the concentration of strontium ions in seawater. The sensor gives a fast and early warning when the scaling conditions are reached. This allows plenty of time to take the necessary measures for scale prevention with minimal cost. The designed sensor has good sensitivity while requiring only 0.2 mL of the test solution. It has an excellent monitoring capability of strontium ion concentrations in seawater with the presence of a large sodium concentration and many other ions. We have recorded a detection level of better than 0.042 mol/L (0.08%) of strontium ions in seawater.

## 2. Materials and Methods

### 2.1. Theoretical Background

We used parametric analysis to investigate the optimal sensitivity level for the designed sensor as a function of permittivity and ion concentrations in different fluids and solutions. Initially, we investigated the sensor response using the relative frequency change $\left(\frac{\Delta f}{f}\right)$ and the sensitivity (*S*) of the sensor to different concentrations of ions using the following equations [26]:

(1) $$\frac{\Delta f}{f}=\frac{\left|f_{0}-f\right|}{f_{0}},$$

(2) $$S=\frac{\Delta f/f}{\Delta \varepsilon }\;\;\;with\;\Delta \varepsilon =\varepsilon_{ref}-\varepsilon ,$$

where *f*_0_ and *f* are the reference solution and the test solution resonance frequencies, respectively, while *ε_ref_* and *ε* are the corresponding reference solution and test solution permittivities, respectively.

For a radiation patch having a rectangular shape, the patch antenna has two fundamental radiation modes. The first is the transverse magnetic (TM) mode TM_010_ with its electrical current along the length direction of the radiation patch, and the second mode is the TM_001_ with its current along the width direction of the patch. Assuming the patch antenna has a perfect ground plane, the resonant frequency (*f_r_*) for the dominant mode can be written as follows [26]:

(3) $$f_{r}=\frac{c}{2L\sqrt{\varepsilon_{r,eff}}}\;,$$

where *c* is the speed of light, *L* is the length of the radiation patch, and *ε_r,eff_* is the effective relative dielectric constant of the substrate medium.

In our design, we consider *W*/*h* ≫ 1, where *W* is the width of the patch and *h* is the thickness of the substrate. In this case, the expression of the effective relative dielectric constant is given by the following expression [26]:

(4) εr,eff=εr+12+εr−121+12hW−12,

where $\varepsilon_{r}$ is the dielectric constant of the substrate material relative to air. For a microstrip line in a rectangular patch with air above the substrate, the values of *ε_r,eff_* are such that 1 ˂ *ε_r,eff_* ˂ *ε_r_*. However, for most applications where *ε_r_* ≫ 1, *ε_r,eff_* will be closer to *ε_r_* of the substrate [26]. In this case, the resonant frequency can be written as:

(5) $$f_{r}=\frac{c}{2L\sqrt{\varepsilon_{r}}}\;,$$

The introduced error in using (5) instead of (3) was estimated to be about 0.15% for a rectangular patch with no slot and similar parameters used in our design.

The resonant frequency *f_r_* is the first characteristic of the microstrip sensor. The calculation of *f_r_*, however, is not as simple for more complex geometries and requires advanced radio frequency (RF) modeling software. The second characteristic of the microstrip antenna is the reflection scattering parameter *S*_11_, which is a representation of the return loss of the microwave excitation signal as a function of frequency. This parameter is given by the following expression [26]:

(6) $$S_{11}=\frac{V^{-}}{V^{+}}\;,$$

where *V*^+^ and *V*^−^ are the voltage levels of the incident and reflected waves, respectively.

### 2.2. Sensor Design and Modeling

In this section, we discuss and present the design parameters of a low-profile microstrip sensor antenna. The sensor design is based on a rectangular radiator with a microstrip feed line. We have selected a single layer antenna design with a rectangular shape for accurate modeling and low manufacturing cost.

**Figure 1:**
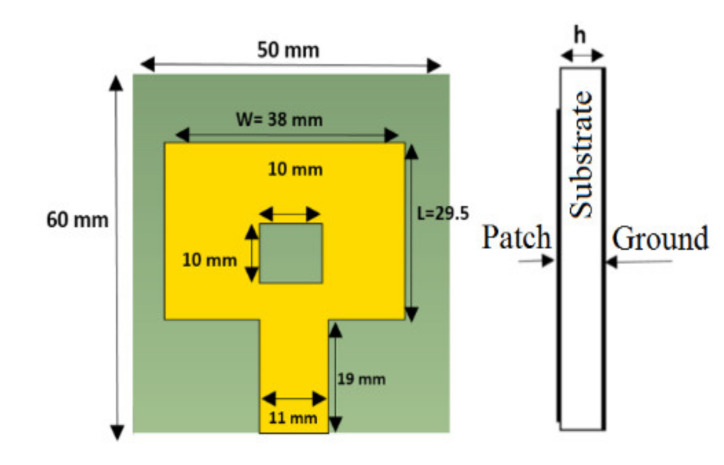
The design parameters of the microstrip sensor used in this study.

Figure 1 shows the final sensor design used for this study. It consists of a rectangular patch with a rectangular slot as the sensing area for probing different ions. We used FR4-epoxy (MG Chemicals, Surrey, Canada) as the substrate with dielectric constant *ε_r_* = 4.205 ± 0.135, thickness *h* = 1.6 mm and loss tangent tanδ = 0.016. The copper layer has a thickness of 66 μm. Part of the patch is the transmission line, which is the rectangular shape where the input signal is connected.

We have modeled the microstrip sensor for operation in the frequency range 3–5 GHz using the CST Microwave Studio software package (Dassault Systèmes, Paris, France). This is done in order to focus on the sensor’s sensitivity at the first resonance frequency located at *f_r_* = 3.87 GHz in the frequency band 3–5 GHz. Several simulations were conducted in order to achieve the lowest bandwidth and reflection coefficient (*S*_11_).

**Figure 2:**
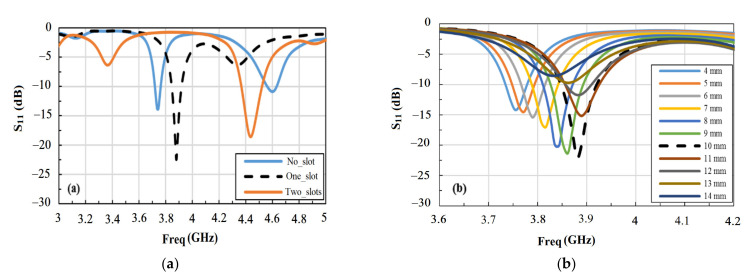
Calculated reflection coefficient for the proposed antenna as a function of the number of slots (**a**) and the size of one slot (**b**) for the radiating element of the microstrip sensor in air.

We have also investigated and optimized the effect of the size and number of slots on the sensor performance. Figure 2 shows the calculated reflection coefficient (*S*_11_) of the sensor as a function of the frequency of the input signal for different design parameters. The simulation results for slot-free, one-slot, and two-slot design were compared as shown in Figure 2a. In particular, we observe the stronger attenuation of the *S*_11_ parameter and the narrower bandwidth at the first resonance frequency in the 3–5 GHz band for one-slot design compared to two-slot and slot-free designs. The signal level decreases from −13.84 dB to −18.45 dB and to −22.39 dB for the radiating element with no slot (slot-free), two slots and one slot, respectively. The stronger the attenuation of *S*_11_, the better the sensitivity of the sensor. The measured full width at half max (FWHM) at the first resonance for one-slot design is about 40 MHz compared to 70 MHz and 160 MHz for the slot-free and the two-slot designs, respectively. For the slot-free design, the performance is lower, and it is only used for comparison as it will not be useful for our application since there is no exposed sensing area. Based on these results, we focus our study on the one-slot design and optimize it further by considering different slot sizes. The results are shown in Figure 2b, which clearly show that the optimum size of the slot is 1 × 1 cm^2^.

The sensor was fabricated according to the optimized sensor design parameters using standard printed circuit board (PCB) processing and etching. In particular, the substrate used was a PCB FR4 Epoxy with the copper layer on both sides of the substrate. The copper thickness, the substrate parameters, dimensions, and design are exactly those presented earlier and shown in Figure 1. Initially, the substrate was cut and etched from the top to have the slotted design shown in Figure 1, while the bottom was kept with a copper layer as the ground of the antenna. After this, an SMA (Sub-Miniature A) coaxial connector (Sihanming technology, Shenzhen, China) was soldered with its ground connected to the ground below the substrate and the signal connected to the feedline on the top of the substrate.

**Figure 3:**
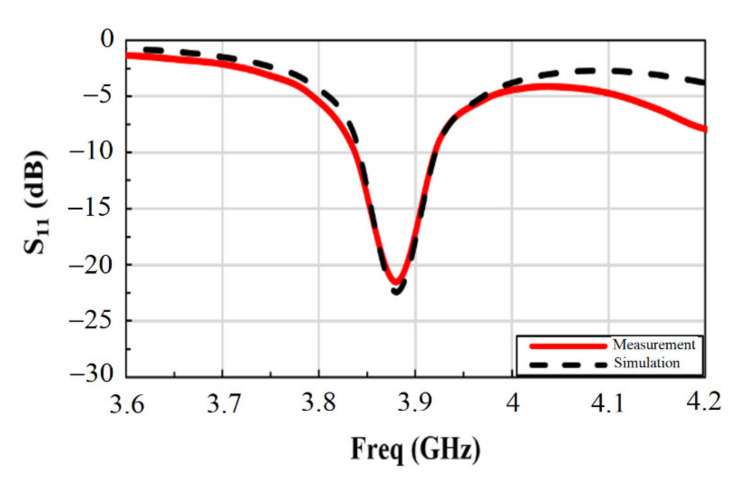
Comparison between the simulated and the measured reflection coefficients of the sensor in air.

The fabricated sensor was tested in air using a vector network analyzer (VNA) (Agilent Technologies, Santa Clara, CA, USA), discussed later in the following section. For comparison, Figure 3 shows the simulated and measured reflection coefficient (*S*_11_) for the first resonance frequency in the 3–5 GHz band using the one-slot design. In particular, we note the good match between the simulation results and the experimental data giving the first resonance peak at 3.88 GHz for air medium. The slight difference observed at higher frequencies could be due to possible discontinuity between the SMA connector and the sensor feedline introduced by the soldering and the fabrication tolerance [27].

**Figure 4:**
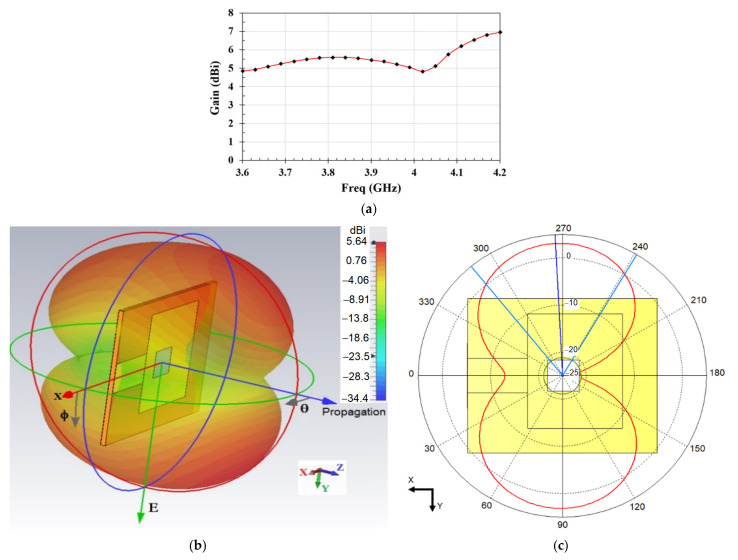
(**a**) Gain of the proposed antenna as a function of frequency in the y-direction. (**b**) Electric (E) field radiation pattern in 3d and (**c**) the E field radiation in the xy plane at the resonant frequency 3.88 GHz in air.

In Figure 4, we show the simulation of the microstrip antenna propagation characteristics at 3.88 GHz in air for reference. The antenna in this study is used as a resonant element that only probes the near field exposed to the strontium solution with an *S*_11_ attenuation coefficient. The antenna is not used as an emitter, which then requires a different experimental configuration allowing for the measurement of *S*_12_ between emitter and receiver antenna. The variation of the gain of the antenna as a function of frequency is shown in Figure 4a, while the radiation pattern is shown in Figure 4b,c. We note in particular that the antenna gain stays relatively large between 4.84 dBi and 6.95 dBi over the operating frequency band considered in this study (3.6–4.2 GHz). In our experiment, however, small gain variations do not affect our measurements since we are using the antenna as a resonant element for probing the resonance frequency shift due to the deposited solution drop on the slot. On the other hand, the radiation pattern shown in Figure 4b,c confirms the full coverage of the electric field interaction with the medium in the slot region.

### 2.3. Experimental Setup and Procedure

In this experiment, we stimulate the sensing area of the sensor using small drops of different solutions, which alter the dielectric constant of the sensor and produce a shift in its resonance frequency. To test the sensor, we used drops of deionized (DI) water, seawater, and strontium nitrate solutions with different strontium concentrations. The drops were applied to the sensing area of the sensor, and the resulting microwave signals were recorded for different microwave excitation frequencies. Preliminary tests have led to the use of four drops with a total of 0.2 mL of the solution as the minimum adequate quantity needed to characterize the frequency response and sensitivity of the sensor. The drop was carefully centered on the exposed area of the sensor with negligible effect due to minor variations in centering the drop on the surface, as can be seen later in the sensor resetting measurements section.

We conducted two sets of experimental tests on the sensor. The first one was the continuity test to obtain the efficiency and sensitivity of the sensor. The second test was the repeatability test to establish the resetting capability of the sensor as well as the sensor lifetime. After each fluid measurement, we dried the sensor and kept it in the air for about 2 min before conducting the next measurement to avoid any sensor contamination.

**Figure 5:**
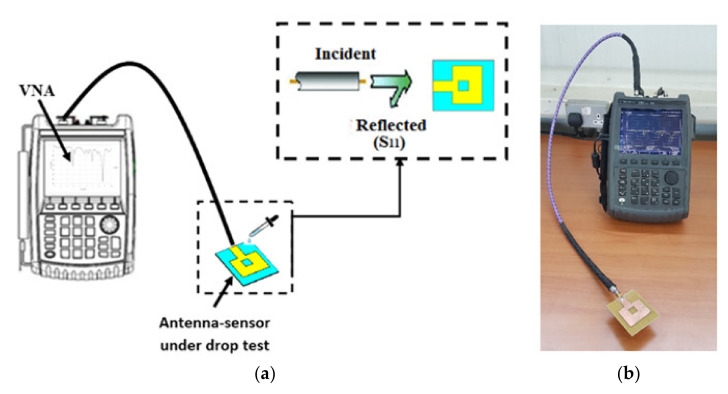
The experimental setup used for the fluid drop-test using the antenna sensor and the VNA to measure the *S*_11_ parameter: (**a**) diagram of the setup and (**b**) actual experimental setup.

The fabricated sensor was connected to the vector network analyzer (VNA) N9918A FieldFox microwave analyzer (Agilent Technologies, Santa Clara, CA, USA). The generated microwave signal at different frequencies was radiated through the sensor antenna, and the reflected waves were detected and recorded simultaneously by the VNA, as shown in Figure 5.

We recorded and compared the resonance frequencies with minimum reflection, i.e., maximum attenuation, and their corresponding magnitudes for different fluids. The difference between the recorded measurements for the different solutions placed on the sensing area of the sensor is related to changes in the substrate dielectric properties. Since the different solutions considered have different dielectric constants, hence they also have different resonant frequencies.

In order to evaluate the sensor sensitivity, the amplitude of the reflection coefficient *S*_11_ and the resonance frequency *f_r_* were measured and recorded as a function of frequency in the 3–5 GHz range. This is mainly probing changes in the first resonance of the sensor in the 3–5 GHz band due to the presence of different fluids on the sensing area of the sensor. The results are presented in the next section.

### 2.4. Strontium Solutions Preparation

Initially, we prepared a supersaturated solution of strontium ions in seawater at room temperature (25 °C) by mixing 53.8 g of strontium nitrate powder (Sigma-Aldrich, St. Louis, MO, USA) into 100 mL of seawater obtained directly from the sea in Abu Dhabi. The solution was stirred and checked to make sure it is saturated. We then prepared standard solutions of strontium in seawater with different concentrations of strontium ions. The first solution was prepared by mixing 10 mL of the prepared saturated strontium solution with 100 mL of seawater. The subsequent solutions were then prepared by adding an additional 10 mL of the saturated strontium solution to the previous solution to increase the strontium concentration. We repeated this process several times to prepare a set of many seawater solutions with gradually increasing concentrations of strontium ions.

## 3. Results and Discussion

### 3.1. Sensor Response to Different Mediums

**Figure 6:**
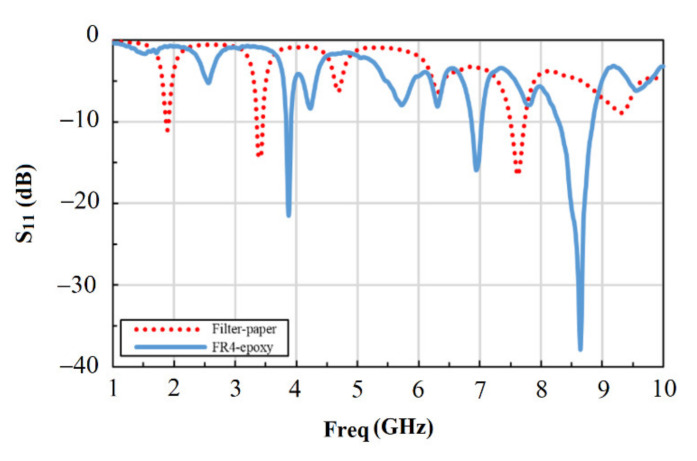
Comparison between the sensor responses in air using two different substrate dielectric materials (filter paper and FR4).

The measurements were initially conducted in air using two sensors with different dielectric substrates for comparison. These two dielectrics are a filter paper and a standard PCB material (FR4). The filter paper was considered for its capacity to absorb the solution and the possibility to enhance the sensitivity. The results in Figure 6 show that the *S*_11_ signal was larger by about 33% for FR4 compared to filter paper at the first resonance in the 3–5 GHz band, while it was even much higher for FR4 at the second resonance in the 7–9 GHz band and reaching about 180% increase. Based on these results, we focus our study on the sensor with FR4 substrate and the frequency range 3–5 GHz for the rest of this paper. This was mainly due to the higher sensitivity of FR4 based sensor and the advantage of reusing it many times compared to the single-use of the paper-based sensor. In addition, the lower frequency range of 3 to 5 GHz was selected for easier probing and simpler future integration with standard electronics.

**Figure 7:**
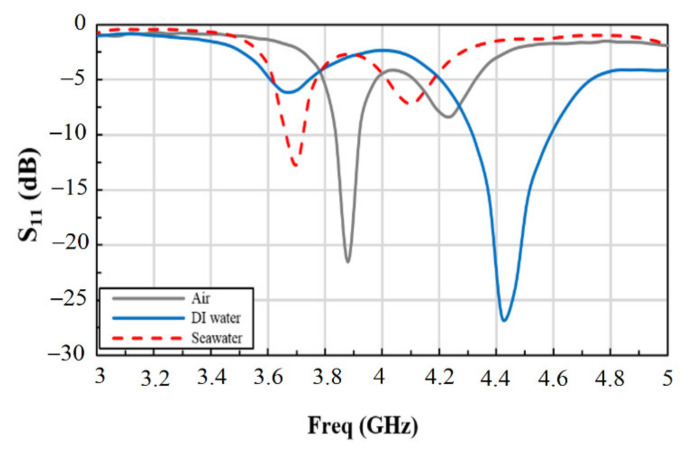
Measured reflection coefficient (*S*_11_) for air, DI water, and seawater as indicated.

Initially, we used FR4 based sensor for measuring its response to different dielectric mediums, which are air, deionized (DI) water, and seawater, as shown in Figure 7 for comparison. The measurements were conducted using a drop with a volume of 0.2 mL of each of these fluids separately deposited in each case on the sensing area of the sensor. This is also referred to later as the “drop-test”. We focused our measurements on the frequency band 3–5 GHz to probe the first resonance. The sensor showed a reflection attenuation coefficient (*S*_11_) of −21.52 dB in air at the resonant frequency of 3.88 GHz and a bandwidth of 85 MHz at −10 dB. On the other hand, DI water and seawater showed resonant frequencies at 3.66 GHz and 3.70 GHz, respectively, with *S*_11_ attenuations of −6.0 dB and −12.7 dB, respectively. From the results, we observed a frequency shift of about 180 MHz between air and seawater, leading to good sensitivity as a function of changes in the dielectric constant.

The change in the fluid dielectric constant from *ε_r_* = 1 (air) to *ε_r_* = 80.1 (DI water) caused a frequency shift of 220 MHz of the resonance frequency. When the sensing area receives a drop of seawater (*ε_r_* = 69), the resonant frequency records a shift of 180 MHz from that of air. We associate the observed large shift of the resonance frequency to the large change in the dielectric constant value for the different fluids. This result confirms that the proposed sensor could be used for probing changes in the resonance frequencies for different fluids and solutions. Theoretically, it is well known that the fractional change in the resonant frequency increases with increasing the dielectric constant of a material [28]. This was observed in our measurements on different fluids (air, DI water, seawater, and strontium solutions). The change in the frequency measured between air and DI water was 5.7%, and between air and seawater was 4.6%. This allows good sensitivity and sensing range. Based on these results, it is expected to be used as a chemical sensor to monitor the concentration of several ions in different liquids with different permittivity and hence benefit from the recent advances in RF technology.

The results from our sensor were compared to similar available data in the literature for air and DI-water. Table 1 shows the comparison of the resonance frequencies and the reported sensitivities from different sources [2,12,14]. In this case, the three sources used a more advanced microfluidic RF-based system for their measurements. As the focus of our sensing capabilities was relative to seawater and strontium concentration, we also show the sensitivity of the sensor relative to the highest strontium solutions in seawater. Based on these results, we confirm that our proposed sensor had comparable sensitivity relative to DI water data available in the literature. However, the sensitivity relative to strontium solutions was shown to be good and was used for investigating the strontium ions monitoring in the seawater environment.

### 3.2. Sensor Resetting Capability

**Figure 8:**
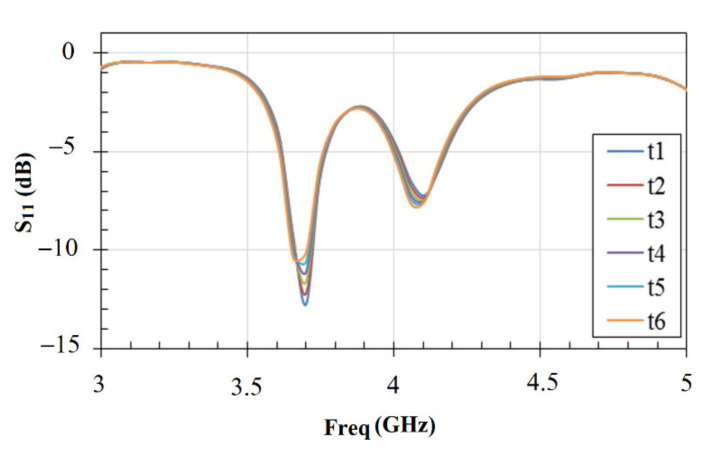
The resetting response of the sensor for the first resonance frequency using seawater as the reference medium (*f_seawater_* = 3.70 GHz) for different times from t1 to t6 at 30 min intervals.

We conducted several experiments to test the sensor resetting capability. According to the obtained results shown in Figure 8, we achieved consistent resetting values of the sensor in seawater after each measurement. The average change recorded for resetting the resonant frequency was about 9 MHz over a band of 300 MHz. These measurements were conducted after the use of each different concentration of strontium ions. The result corresponds to about 3% overall resetting error and an average change in the attenuation level of *S*_11_ of about −0.47 dB. According to our calibration of the sensor, the 9 MHz frequency shift corresponded to the strontium ions concentration of 0.04 mol/L (0.08%). This small variation also included any minor effect related to centering the drop each time on the sensing area of the sensor, as discussed above.

### 3.3. Strontium Ions Concentration Monitoring in Seawater

**Figure 9:**
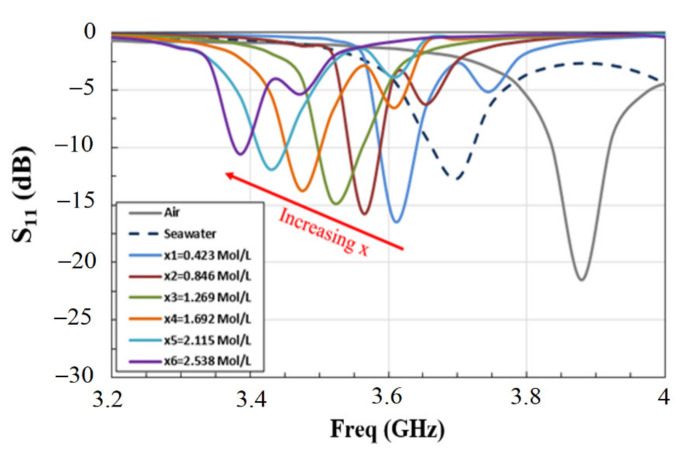
Measured reflection coefficient (*S*_11_) of the proposed sensor for solutions with different strontium concentrations (x) in seawater as indicated.

In this section, we focus our attention on investigating the sensor’s capabilities of monitoring the concentration of strontium ions in seawater. The measurements were conducted using the proposed sensor and experimental setup on the previously prepared strontium solutions with different strontium concentrations. Before each measurement, we stirred the solution to ensure it was homogeneous. We conducted our measurements by placing a 0.2 mL drop of the solution on the active area of the sensor and recording the obtained RF signal. We repeated this process for each strontium concentration, and the results are shown in Figure 9. From the figure, we can clearly observe a significant shift of the resonance frequency as a function of increased strontium ions concentration (x) in seawater. The initial frequency was 3.7 GHz for seawater alone with no strontium ions added (x = 0). By increasing the concentration x of the strontium ions, we observed a strong decrease in the resonance frequency. After reaching the maximum concentration with x = 2.538 mol/L of strontium ions in seawater, the resonance frequency became 3.39 GHz. This corresponded to a large frequency shift of 315 MHz, which gave a relative frequency shift of 8.5%. This result was an indication of the good sensitivity of the sensor for applications in strontium concentration monitoring.

**Figure 10:**
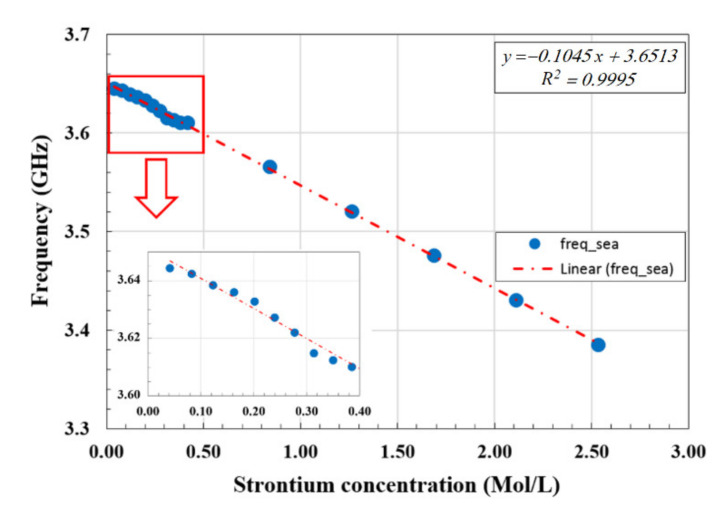
The resonance frequency shifts as a function of strontium concentration in seawater, showing a good linear fit. The inset shows the magnified region with the smallest concentrations.

Based on these results, we can confirm that the sensor showed a good frequency response to small changes in the material dielectric constant. These changes in the dielectric constant were attributed to the small changes in the concentrations of strontium ions in seawater. The frequency shifts Δ*f* observed for the resonance frequency at different concentrations of strontium ions are shown in Figure 10. In particular, we note the good linear relation obtained between the resonance frequency and the strontium ions concentration with the R^2^ fitting factor of 99.95%. This confirms the high sensing capability and the potential as a strontium ion monitoring sensor.

In addition, we conducted further measurements to identify the smallest strontium ions concentration measurable by the sensor. The results of these measurements are shown in the inset of Figure 10. The lowest and well-resolved strontium ions concentration that we were able to measure was 0.042 mol/L. We also note that even at these low concentrations, the sensor response was still showing the same good linear fit as in the high concentrations.

The observed decrease in the resonance frequency of the microstrip sensor as the strontium ions concentration increased partly due to the reduced mobility of the water molecules in the solution due to the presence of strontium ions. On the other hand, the degree of impedance mismatch between the microstrip antenna sensor operating in air medium was also reduced due to the presence of the fluid samples on the microstrip substrate. Similarly, the impedance mismatch was reduced further with the increased concentration of strontium ions on the microstrip substrate, hence reducing the magnitude of the *S*_11_ signal at the resonance frequency, as shown in Figure 9. This was also consistent with the reduced number of free water molecules as the strontium ions concentration increased [29].

**Figure 11:**
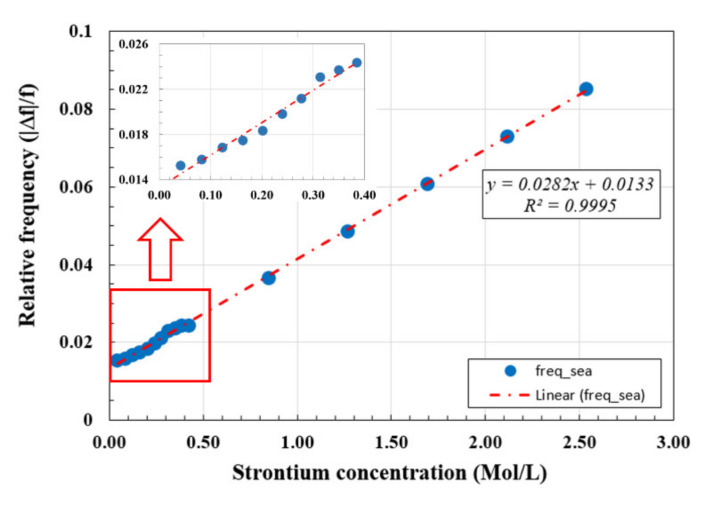
Relative frequency as a function of strontium ions concentration (x) in seawater. The dash-dotted line is the linear fit to the data, and the inset is the expansion of the small strontium concentration region as indicated by the box.

The relative resonance frequency change and the sensing capabilities of the sensor are investigated using seawater as the reference in (1) with $f_{seawater}=3.70\;\mathrm{GHz}$ as shown in Figure 8. In Figure 11, we show the variation of the relative frequency (Δ*f*/*f*) as a function of strontium ions concentration x in seawater. As can be observed, a good linear relation was obtained between the magnitude of the relative resonance frequency and the concentration of strontium ions. The magnitude of the relative frequency increased as the strontium ions concentration in seawater increased. This provides good sensing and monitoring capabilities of the concentration of strontium ions in seawater environment, which contains many other ions, especially sodium (Na^+^).

The obtained linear relation between the resonance relative frequency and the strontium ions concentration can be related to the relative change in the dielectric constant of the material on the microstrip sensor substrate. The results in Figure 11 show a good linear fit to the data using (7) with the R^2^ fitting parameter of 99.95%:

*y* = 0.0282 x + 0.0133, (7)

where *y* is the magnitude of the relative frequency compared to seawater and x is the strontium ions concentration in seawater in mol/L. This shows the good sensing capability of the sensor and a well-established resetting property over a long time period. In addition, the amount of fluid required for this sensitivity was small and consisted of a fluid drop volume of only 0.2 mL. The material used for the sensor makes it more robust and can be used many times in different fluids, while it is also expected to work well in harsh environments.

This is an important result, which has many applications, especially in offshore oil production for scale monitoring. In addition, our results could support future studies on monitoring the concentration levels of other ions such as calcium (Ca^++^), which causes challenging problems related to scaling in steam pipes and water boilers.

## 4. Conclusions

We report the design and performance of a GHz sensor for the successful monitoring of the concentration of strontium ions in seawater, which contains many other ions such as sodium, chlorine, magnesium and calcium. The sensor showed a large frequency range for measurements consisting of about 1035 MHz. This range covers measurements in both air, DI water, seawater, and seawater with different concentrations of strontium ions. We have demonstrated the high sensitivity of the GHz microstrip sensor to monitor the concentration of strontium ions in seawater with values as low as ±0.04 mol/L. The sensor only requires a small drop of the solution with a volume of 0.2 mL. In comparison to the available results in the literature, we have conducted measurements using DI water and confirmed the good sensing capability of our sensor. On the other hand, the current sensor is robust and reusable for many times without any cross-contamination. In the future, this work could be extended further to investigate the simultaneous monitoring of multiple ions with enhanced sensor design and sensitivity.

## Figures and Tables

**Table 1:** Some reported resonance frequencies and sensitivities using air and deionized (DI) water for comparison.

Reference	*f*_0_ (GHz)	*f* (GHz)	Sensitivity (×10^−3^)
[2] ^1^	0.92	1.00	1.09
[12] ^1^	1.18	1.52	3.60
[14] ^1^	3.07	3.38	1.26
This work ^1^	3.88	3.66	0.72
This work ^2^	3.88	3.39	1.86

^1^ References using RF/microwave methods with *ε_r,air_* = 1 and *ε_r,water_* = 80.1. ^2^ For the highest strontium concentration solution with *ε_r,air_* = 1 and *ε_r,seawater_* = 69.

## Data Availability

The data presented in this study are available on request from the corresponding author.

## References

[1] Gregory A., Clarke R. (2006). A review of RF and microwave techniques for dielectric measurements on polar liquids. IEEE Trans. Dielectr. Electr. Insul..

[2] Mariotti C., Su W., Cook B.S., Roselli L., Tentzeris M.M. (2015). Development of Low Cost, Wireless, Inkjet Printed Microfluidic RF Systems and Devices for Sensing or Tunable Electronics. IEEE Sens. J..

[3] Salim A., Lim S. (2018). Simultaneous detection of two chemicals using a TE_02_ Quarter-Mode Substrate-Integrated Waveguide Resonator. Sensors.

[4] Hossain A., Islam M.T., Islam M.T., Chowdhury M.E.H., Rmili H., Samsuzzaman M. (2020). A Planar Ultrawideband Patch Antenna Array for Microwave Breast Tumor Detection. Materials.

[5] Ibanez-Labiano I., Alomainy A. (2020). Dielectric Characterization of Non-Conductive Fabrics for Temperature Sensing through Resonating Antenna Structures. Materials.

[6] Van Baelen D., Lemey S., Verhaevert J., Rogier H. (2018). A Novel Manufacturing Process for Compact, Low Weight and Flexible Ultra-Wideband Cavity Backed Textile Antennas. Materials.

[7] Galindo-Romera G., Herraiz-Martínez F.J., Gil M., Martínez-Martínez J.J., Segovia-Vargas D. (2016). Submersible Printed Split-Ring Resonator-Based Sensor for Thin-Film Detection and Permittivity Characterization. IEEE Sens. J..

[8] Chahadih A., Cresson P.Y., Hamouda Z., Gu S., Mismer C., Lasri T. (2015). Microwave/Microfluidic Sensor Fabricated on a Flexible Kapton Substrate for Complex Permittivity Characterization of Liquids. Sens. Actuators A.

[9] Eom S., Lim S. (2016). Stretchable Complementary Split Ring Resonator (CSRR)-Based Radio Frequency (RF) Sensor for Strain Direction and Level Detection. Sensors.

[10] Horestani A.K., Naqui J., Shaterian Z., Abbott D., Fumeaux C., Martín F. (2014). Two-Dimensional Alignment and Displacement Sensor Based on Movable Broadside-Coupled Split Ring Resonators. Sens. Actuators A.

[11] Lee H., Lee J., Moon H., Jang I., Choi J., Yook J., Jung H. (2012). A Planar Split-Ring Resonator-Based Microwave Biosensor for Label-Free Detection of Biomolecules. Sens. Actuators B.

[12] Ebrahimi A., Withayachumnankul W., Al-Sarawi S.F., Abbott D. Microwave microfluidic sensor for determination of glucose concentration in water. Proceedings of the IEEE 15th Mediterranean Microwave Symposium.

[13] Gennarelli G., Romeo S., Scarfì M.R., Soldovieri F. (2013). A Microwave Resonant Sensor for Concentration Measurements of Liquid Solutions. IEEE Sens. J..

[14] Jankovic N., Radonic V. (2017). A Microwave Microfluidic Sensor Based on a Dual-Mode Resonator for Dual-Sensing Applications. Sensors.

[15] Vaks V. (2012). High-Precise Spectrometry of the Terahertz Frequency Range: The Methods, Approaches and Applications. J. Infrared Millim. Terahertz Waves.

[16] Eldamak A.R., Fear E.C. (2018). Conformal and Disposable Antenna-Based Sensor for Non-Invasive Sweat Monitoring. Sensors.

[17] Merdhah A., Yassin A. (2009). Scale Formation Due to Water Injection in Malaysian Sandstone Cores. Am. J. Appl. Sci..

[18] Bouchalkha A., Karli R., Alhammadi K., Anjum A., Saadat I., Al Ghaferi A. Graphene Based Sensor for Scale Monitoring. Proceedings of the RDPETRO 2018: Research and Development Petroleum Conference and Exhibition.

[19] Bouchalkha A., Karli R., Alhammadi K. Graphene Sensor for Scale Monitoring Applications in Oil Pipelines. Proceedings of the 2020 Advances in Science and Engineering Technology International Conferences (ASET).

[20] Temizel C., Thanon D., Inceisci T., Balaji K., Suhag A., Ranjith R., Wijaya Z., Abdelfatah E. An Analysis of Scale Build-up in Seawater Injection of Water Flooding Operations. Proceedings of the SPE Latin America and Caribbean Mature Fields Symposium.

[21] Al-Matar H., Al-Ashhab J.K., Mokhtar S.R.M. (2006). Techniques Used to Monitor and Remove Strontium Sulfate Scale in UZ Producing Wells. Soc. Pet. Eng..

[22] Crabtree M., Eslinger D., Fletcher P., Miller M., Johnson A., King G. (1999). Fighting scale-removal and prevention. Oilfield Rev..

[23] Bamidele O.A., Falode O.A., Omole O. (2009). Effects of Oil Field Scale Deposition on Oil Production from Horizontal Wells. Pet. Coal.

[24] Alhammadi K., Bouchalkha A., Sowwan S. Scale detection using light sensing technique. Proceedings of the 2015 IEEE Jordan Conference on Applied Electrical Engineering and Computing Technologies (AEECT).

[25] Amiri M., Moghadasi J., Jamialahmadi M. (2014). A Prediction of the Amount of Strontium Sulfate Scale Formation in Siri Oilfield at Different Temperatures and Pressures. Energy Sources Part A Recovery Util. Environ. Eff..

[26] Balanis C.A. (2016). Antenna Theory: Analysis & Design.

[27] Karli R., Ammor H., Therzaz J., Chaibi M., Sanchez A.M. (2015). Design and construction of miniaturized UWB microstrip antenna with slots for UWB applications. Microw. Opt. Technol. Lett..

[28] Chen L.F., Ong C.K., Neo C.P., Varadan V.V., Varadan V.K. (2004). Microwave Electronics: Measurement and Materials Characterization.

[29] Rahman M.N., Islam M.T., Sobuz M.S. (2018). Microwave measurement system to detect salt and sugar concentration. Microw. Opt. Technol. Lett..

